# The computerized algorithm for renal assessment improves diagnostic accuracy of renal impairment in hospitalized patients

**DOI:** 10.1038/s41598-025-87424-7

**Published:** 2025-01-31

**Authors:** Chun-You Chen, Te-I. Chang, Cheng-Hsien Chen, Shih-Chang Hsu, Yen-Ling Chu, Nai-Jen Huang, Yuh-Mou Sue, Tso-Hsiao Chen, Po-Hsun Huang, Chung-Te Liu, Hui-Ling Hsieh

**Affiliations:** 1 Department of Radiation Oncology, Wan Fang Hospital, Taipei Medical University, Taipei, Taiwan; 2 Department of Surgery, School of Medicine, College of Medicine, Taipei Medical University, Taipei, Taiwan; 3 Division of Cardiovascular Surgery, Department of Surgery, Wan Fang Hospital, Taipei Medical University, Taipei, Taiwan; 4 Graduate Institute of Biomedical Electronics and Bioinformatics, National Taiwan University, Taipei, Taiwan; 5 Department of Internal Medicine, School of Medicine, College of Medicine, Taipei Medical University, Taipei, Taiwan; 6 Division of Nephrology, Department of Internal Medicine, Wan Fang Hospital, Taipei Medical University, Taipei, Taiwan; 7 Division of Nephrology, Department of Internal Medicine, Shuang Ho Hospital, Taipei Medical University, Taipei, Taiwan; 8 Emergency Department, Department of Emergency and Critical Medicine, Wan Fang Hospital, Taipei Medical University, Taipei, Taiwan; 9 Department of Emergency Medicine, School of Medicine, College of Medicine, Taipei Medical University, Taipei, Taiwan; 10 Division of Cardiology, Department of Medicine, Taipei Veterans General Hospital, Taipei, Taiwan; 11 Second Degree Bachelor of Science in Nursing Collage of Medicine, National Taiwan University, Taipei, Taiwan; 12 Department of Nursing, National Taiwan University Hospital Yunlin Branch, Yunlin, Taiwan

**Keywords:** Acute kidney injury (AKI), Chronic kidney disease (CKD), Intensive care unit (ICU), Hospitalization, Medical research, Nephrology

## Abstract

In hospitalized patients, acute kidney injury (AKI) is an important adverse event associated with high mortality and medical costs. Accurate diagnosis and timely management of AKI are essential for improving the outcomes of in-hospital AKI, and delayed diagnosis or misdiagnosis hinders advancements in AKI care. To ameliorate this problem, several electronic AKI alert systems have been proposed but have shown inconsistent effects on AKI outcomes. Before electronic systems can improve AKI outcomes, it is important to confirm their diagnostic accuracy. The purposes of the present study were to establish an easy-to-construct computerized algorithm for the diagnosis of renal impairment and to test its accuracy. The present study retrospectively included 1551 patients hospitalized in Wanfang Hospital with serum creatinine (SCr) levels > 1.3 mg/dL. A computerized algorithm was constructed to identify AKI events and chronic kidney disease (CKD) in these patients. Previous SCr tests were reviewed to define baseline SCr levels. A SCr level increased > 1.5 times from baseline was defined as AKI. An estimated glomerular filtration rate (eGFR) of < 60 mL/min/1.73 m^2^ for > 90 days was defined as CKD. Discharge diagnoses made by the attending physicians were defined as “clinician’s diagnoses.” The researcher’s diagnoses, made by experienced nephrologists according to the same criteria, were the gold standard to which the computerized algorithms and the clinician’s diagnoses were compared. The diagnoses made by the computerized algorithm and clinician were compared with the researcher’s diagnoses to define their accuracy. Among the included patients, the mean age was 73.0 years; in-hospital mortality was 14.8%, and AKI was present in 28.6% of patients. Regarding the diagnostic accuracy for AKI, the computerized algorithm achieved a sensitivity of 85.6% and a specificity of 98.8%. The main cause of false-negative (FN) AKI diagnosis was AKI occurring prior to the outpatient visit, before the indexed hospitalization. Regarding the diagnostic accuracy for CKD, the computerized algorithm achieved a sensitivity of 94.7% and specificity of 100%. The main cause of FN CKD diagnosis was the lack of previous eGFR records. The computerized algorithm exhibited significantly superior accuracy compared to the clinician’s diagnoses for both AKI (95.0% vs. 57.0%) and CKD (96.5% vs. 73.6%). We developed a simple and easy-to-construct computerized algorithm for the diagnosis of renal impairment that demonstrated significantly improved diagnostic accuracy for AKI and CKD compared to that of clinicians. In the future, an algorithmic approach for the differential diagnosis of AKI and a decision guide should be incorporated into this system.

## Introduction

Acute kidney injury (AKI) is one of the most common complications among hospitalized patients^1^, is associated with high in-hospital mortality and long hospital stays^2^. If not treated appropriately, AKI may become irreversible and progress to chronic kidney disease (CKD) or end-stage renal disease (ESRD)^3^. In official reports, the annual cost associated with AKI was as high as £1 billion in the United Kingdom and $5.4–$24.0 billion in the United States, causing a heavy burden on the healthcare system^4,5^. Early and accurate diagnosis is one of the most accepted strategies for attenuating the medical burden associated with AKI^6,7^.

Although the criteria for AKI are clearly defined by clinical guidelines, it is often overlooked by clinicians, which increases medical costs and mortality among hospitalized patients^5,8^. One solution to improve the diagnosis of AKI is to use an electronic AKI alert system. In a previous study conducted in intensive care units (ICUs), an electronic AKI alert system significantly improved renal function^9^. In another multicenter study of hospitalized patients, a computerized clinical-decision support system resulted in small decreases in hospital mortality, dialysis use, and length of hospital stay^10^. The results of these studies suggest that computerized or electronic AKI alert systems may be a solution for improving in-hospital AKI outcomes. However, this approach has certain drawbacks. In an observational cohort study with segmented regression analysis conducted in the United Kingdom, implementation of an electronic AKI alert system and AKI-related staff education did not reduce the severity or mortality associated with AKI^11^. In a previous study conducted in Korea, although the implementation of electronic AKI alerts with automated nephrologist consultations improved the likelihood of AKI recovery, the mortality of patients with AKI was not affected^12^. In another multicenter trial involving electronic AKI alert systems, AKI care bundles, and educational programs, the incidence of AKI paradoxically increased, and 30-day AKI mortality was not reduced^13^. To date, whether electronic AKI alert systems with automated nephrologist consultation or AKI care bundles improve the outcome of AKI remains unclear^14–16^.

While the early recognition of AKI is beneficial, the electronic AKI alert system has not been shown to improve AKI outcomes. One reason for this disappointing result is that electronic AKI alert systems do not change the clinical management of AKI^11^. Frequent automatic alerts are likely to cause alert fatigue, which means clinicians become desensitized to the alerts and fail to response effectively^17^. In addition, the uncertain accuracy of the electronic AKI alert system hinders its application in clinical practice. In our experience, electronic AKI alert systems often provide excessive alerts containing CKD or ESRD results that do not require immediate evaluation. To avoid this, an electronic AKI alert system must provide accurate and streamlined alerts. The accuracy of electronic AKI alert systems, however, has not been validated in previous studies, rendering it infeasible to evaluate their real effect on the outcomes of patients with AKI.

To compensate for this gap, the present study aimed to develop and test the accuracy of a computerized algorithm for the assessment of renal impairment that is capable of accurately classifying renal impairment data into the diagnosis of AKI or CKD, providing correct AKI alerts, and avoiding overabundant messages to clinicians.

## Material and methods

### Study design and participants

The study was conducted at Wanfang Hospital, Taipei Medical University. Medical records of patients hospitalized between March 2020 to December 2020 were retrospectively reviewed. All patients older than 20 years with at least one record of a serum creatinine (SCr) level > 1.3 mg/dL were included in the analysis. The “clinicians’ diagnoses” of AKI or CKD were made independently by the attending physician or surgeon and documented in discharge summaries. Remarkably, the clinicians’ diagnoses were made without further validation, further demonstrating the need to verify the accuracy of such diagnoses. The series of SCr data from more than 90 days before admission to the end of hospitalization was interpreted using the computerized algorithm established in this study to automatically generate the diagnoses. The “researcher’s diagnosis,” made by two experienced nephrologists based on a review of the same series of laboratory data, was the gold standard to which the computerized algorithm and clinician’s diagnoses were compared to define their accuracy. The present study was approved by the Ethics Committee and Institutional Review Board of Taipei Medical University (N202111017). All the participants provided informed consent. This study was conducted in accordance with the tenets of the 1975 Declaration of Helsinki, revised in 2000.

### Researcher’s definitions for AKI and CKD

The definition of AKI was adapted from the Kidney Disease: Improving Global Outcomes (KDIGO) Clinical Practice Guideline for AKI. As an abnormal SCr value was identified (> 1.3 mg/dL), data from previous SCr tests were reviewed. AKI was defined in the following situations: 1. For those who underwent previous SCr tests within 7 days before the indexed abnormal SCr values, an increase in the SCr value > 1.5 was defined as AKI. However, AKI was not observed. 2. For those who had previous SCr tests more than 7 days before the indexed abnormal SCr values, the nearest previous SCr value was assumed to be the baseline SCr level, and an increase in SCr value of > 1.5 times this level was defined as AKI. Otherwise, AKI was not observed. 3. For those without any previous SCr tests, the patient was assumed to have a normal baseline SCr level, and AKI was defined as present. Notably, in the present study, the AKI criterion of decreased urine output in the KDIGO Clinical Practice Guidelines was not adopted because the documentation of urine output was unconfirmed.

The definition of CKD was adapted from the KDIGO Clinical Practice Guideline for CKD. The estimated glomerular filtration rate (eGFR) was calculated using the CKD Epidemiology Collaboration (CKD-EPI) equation^18^. If an abnormal eGFR was identified (< 60 mL/min/1.73 m^2^), the data of the previous eGFR were reviewed. CKD was defined in the following situations: 1. For those with abnormal eGFR values > 90 days before the indexed abnormal eGFR value, CKD was defined as present. 2. For those with normal eGFR values > 90 days before the indexed abnormal eGFR values, CKD was defined as absent. Notably, the CKD criteria for ultrasonographic findings and proteinuria in the KDIGO Clinical Practice Guidelines were not adopted because these examinations were not routinely performed; thus, these data were not generally available for the computerized algorithms.

### Programmatic approach for the diagnosis of renal impairment

**Fig. 1:**
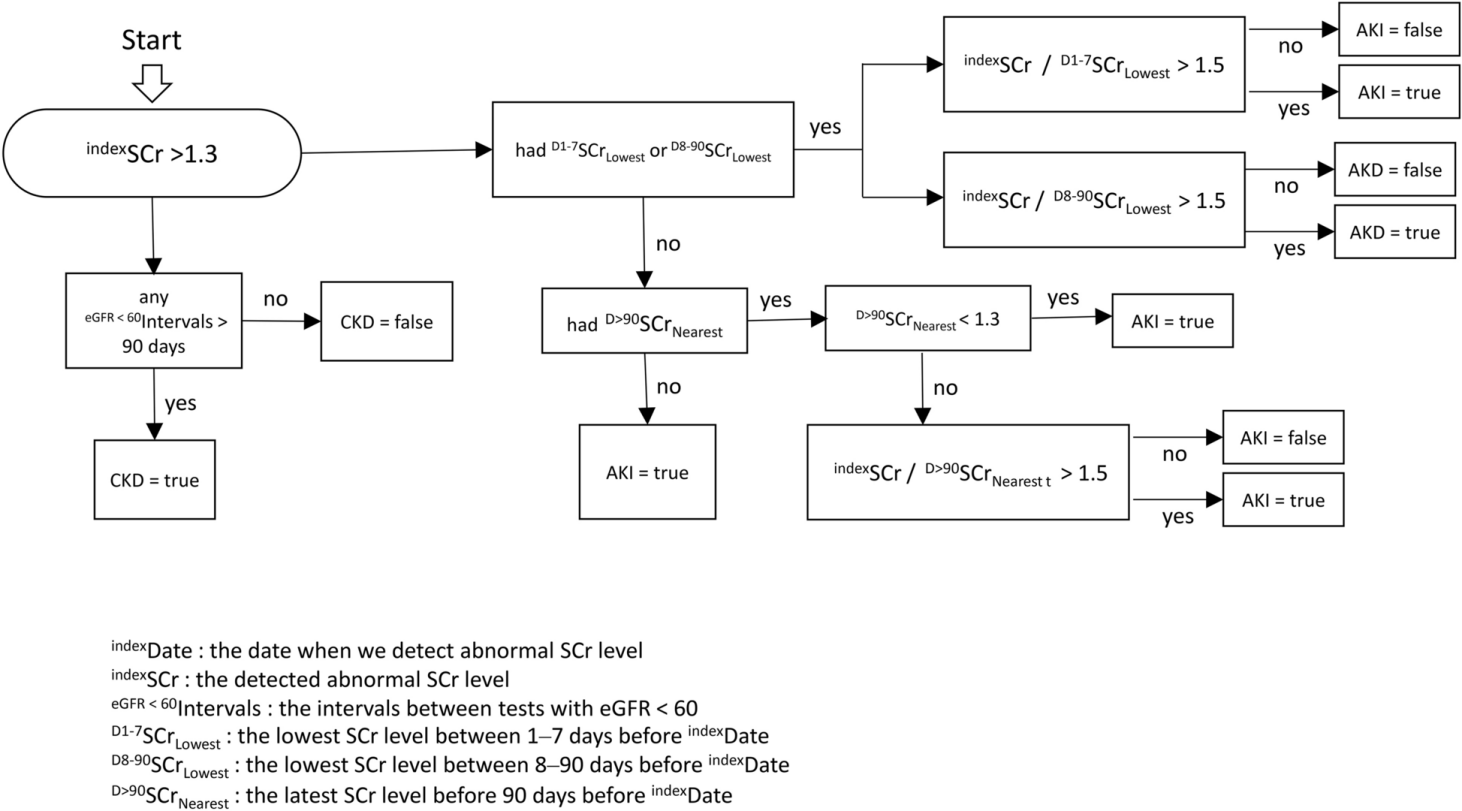
Algorithm chart. ^index^Date, the date when we detect abnormal SCr level; ^index^SCr, the detected abnormal SCr level; ^eGFR < 60^Intervals, the intervals between tests with eGFR < 60; ^D1-7^SCr_Lowest_, the lowest SCr level between 1 and 7 days before ^index^Date; ^D8-90^SCr_Lowest_, the lowest SCr level between 8 and 90 days before ^index^Date; ^D>90^SCr_Nearest_, the latest SCr level before 90 days before ^index^Date. eGFR, estimated glomerular filtration rate; SCr, serum creatinine.

The program code was edited using Node.js 14.19.1 (OpenJS Foundation, San Francisco, CA, USA). For cases in which an abnormal SCr level (> 1.3 mg/dL) was identified by the program, the presence of AKI or CKD was defined independently. For cases in which the patient had record of a previous SCr test within 90 days, AKI was defined as an increase in the SCr value of > 1.5 times the baseline value. For cases in which there were no records of previous SCr testing in the database, the patient was assumed to have a normal baseline SCr, and AKI was defined as present. For cases in which there were records of SCr testing > 90 days prior, the previous SCr value was assumed to be the baseline, and the presence of AKI was defined as an increase in SCr of > 1.5 times this value. For case in which there were records of eGFR < 60 mL/min/1.73 m^2^ more than 90 days prior, CKD was defined as present (Fig. 1). The pseudocode for the algorithm is provided as a supplementary file.

### Statistical analyses

Continuous variables with normal distribution were expressed as mean ± standard deviation (SD); continuous variables that deviated from normal distribution were expressed as medians and interquartile ranges; categorical variables were expressed as frequencies and percentages. Statistical tests for continuous variables with normal distribution were performed using a two-tailed t-test for unpaired samples. Statistical tests for continuous variables deviating from normal distribution were performed using non-parametric methods. Statistical tests for categorical variables were performed using the chi-squared test. *P* < 0.05 was defined as significant. Diagnostic accuracy was evaluated using a diagnostic table. Statistical analyses were performed using SAS 9.4 (SAS Institute Inc., Cary, NC, USA).

## Results

### Characteristics of the study subjects

During the 10-month study period, 1551 hospitalized patients with at least one record of a SCr level > 1.3 mg/dL were included in the analyses. Of all the included patients, the mean age was 73.0 (SD 14.9) years, 63.1% were male, in-hospital mortality was 14.8%, and AKI was present in 28.6%. Regarding the admission departments, 70.9% were admitted to medical services, and 29.1% were admitted to nonmedical services. Regarding the source of patients, 56.6% were admitted from the emergency department (ED), and 43.4% were admitted from the outpatient department (OPD). Compared to patients admitted from the OPD, those admitted from the ED were significantly older and had significantly higher mortality and more AKI events. Additionally, patients admitted to the ED had significantly more cases of heart failure, atrial fibrillation, dementia, and acute stroke. In contrast, patients admitted from the OPD had significantly more cases of dyslipidemia and solid cancer. Notably, the percentages of patients with CKD, hypertension, coronary artery disease, and diabetes mellitus were similar among the patients from different sources (Table 1).

**Table 1:** Characteristics of the included hospitalized patients with renal impairment.

Demographics	Total patients	Emergency source	Outpatient source	*P* values
Number, n (%)	1551 (100.0)	878 (56.6)	673 (43.4)	n/a
Male, n (%)	978 (63.1)	511 (58.2)	467 (69.4)	< .001
Age, years	73.0 ± 14.9	76.2 ± 15.3	68.7 ± 13.1	< .001
Medical service, n (%)	1100 (70.9)	774 (88.2)	326 (48.4)	< .001
Mortality, n (%)	230 (14.8)	199 (22.7)	31 (4.6)	< .001
AKI (Researcher defined), n (%)	443 (28.6)	327 (37.2)	116 (17.2)	< .001
Comorbidities				
Heart failure, n (%)	235 (15.2)	168 (19.1)	67 (10.0)	< .001
AFib, n (%)	168 (10.8)	111 (12.6)	57 (8.5)	< .001
Dementia, n (%)	146 (9.4)	133 (15.2)	13 (1.9)	< .001
CKD, n (%)	674 (43.5)	383 (43.6)	291 (43.2)	.880
Hypertension, n (%)	1161 (74.9)	646 (73.6)	515 (76.5)	.185
Dyslipidemia, n (%)	474 (30.6)	242 (27.6)	232 (34.5)	.003
CAD, n (%)	413 (26.6)	224 (25.5)	189 (28.1)	.256
Stroke, n (%)	276 (17.8)	202 (23.0)	74 (11.0)	< .001
DM, n (%)	703 (45.3)	417 (47.5)	286 (42.5)	.050
Solid cancer, n (%)	436 (28.1)	179 (20.4)	257 (38.2)	< .001
Lymphoma/Leukemia, n (%)	41 (2.6)	21 (2.4)	20 (3.0)	.480
Gout, n (%)	207 (13.4)	116 (13.2)	91 (13.5)	.858
Tuberculosis, n (%)	39 (2.5)	25 (2.9)	14 (2.1)	.338
Virus hepatitis, n (%)	118 (7.6)	60 (6.8)	58 (8.6)	.189

AKI, acute kidney injury; AFib, atrial fibrillation; CKD, chronic kidney disease; CAD, coronary artery disease; DM, diabetes mellitus; n/a, not applicable.

Continuous variables were expressed in mean ± standard deviation and analyzed using student’s t test for independent samples; categorical variables were expressed as number (%) and analyzed using chi square test.

The median hemoglobin level was 10.6 g/dL, that of the blood urea nitrogen (BUN) level was 41 mg/dL, and that of the SCr level was 2.2 mg/dL. Patients with AKI had significantly higher white blood cell counts, lower platelet counts, higher BUN levels, and higher aspartate and alanine aminotransferase levels than those without AKI. Notably, the SCr levels in patients with AKI were not significantly higher than those in patients without AKI. Patients with AKI during hospitalization had higher mortality and dialysis rates than those without AKI. Among the patients with AKI, 13.0% had a known history of CKD. Interestingly, among the non-AKI patients with CKD, only 52.8% had documented history of CKD, indicating that clinicians may not have been able to thoroughly evaluate CKD history of the hospitalized patients. These findings suggest that, while patients with AKI showed higher disease severity and mortality, their SCr levels and eGFRs were similar to those of patients without AKI at the time of admission (Table 2).

**Table 2:** Laboratory profiles of the included hospitalized patients with renal impairment.

Characteristics	Total	AKI absent	AKI present	*P* values
	N = 1551	N = 1108	N = 443	
WBC, 10^3^/μL	7.7 (5.4)	7.2 (4.4)	10.1 (7.9)	< .001
Hemoglobin, g/dL	10.6 (3.5)	10.6 (3.5)	10.5 (3.6)	.865
Platelet, 10^3^/μL	186 (111)	192 (109)	165 (125)	< .001
Na, mmol/L	138 (6)	138 (5)	138 (10)	.008
K, mmol/L	4.2 (0.8)	4.2 (0.8)	4.2 (0.9)	.713
BUN, mg/dL	41 (40)	38 (36)	48.5 (53.0)	< .001
Creatinine, mg/dL	2.2 (2.6)	2.1 (3.0)	2.5 (2.1)	.076
AST, U/L	21 (15)	19 (12)	26 (31)	< .001
ALT, U/L	16 (16)	15 (14)	20 (26)	< .001
Known CKD history	674(43.5%)	586(52.8%)	88(19.8%)	< .001
Dialysis	311(20.0%)	240(21.6%)	71(16.0%)	.014
Mortality, n (%)	230 (14.8)	85 (7.7)	145 (32.7)	< .001

AKI, acute kidney injury; WBC, white blood cell; BUN, blood urea nitrogen; AST, aspartate aminotransferase; ALT, alanine aminotransferase.

Continuous variables were expressed in median (interquartile range) and were compared using Wilcoxon Rank Sum Test. Category variables were expressed in number (percentage) and were compared using chi square test.

### Diagnostic accuracy of computerized algorithm for AKI

To define the actual AKI events in the present study, the complete series of SCr data was reviewed by researchers who were exclusively nephrology specialists. Any increase of SCr level to ≧1.5 times baseline within the prior 7 days defined AKI, criteria adapted from the KDIGO Clinical Practice Guideline for AKI. For those who did not have baseline SCr levels within the previous 7 days, the nearest previous SCr level was assumed to be the baseline. For those who had no record of prior SCr levels, the baseline SCr level was assumed to be normal. For a single hospitalization course, more than one episode of AKI events were considered “a hospitalization with AKI event.” Notably, in contrast to the researcher’s definition of AKI, the computerized algorithm used the highest SCr level during hospitalization as the indexed SCr level for possible AKI. An increase in SCr level > 1.5 times the previous reading defined hospitalization with an AKI event.

Of the included 1551 patients with at least one record of a SCr level > 1.3 mg/dL, 443 were defined as having AKI events during the indexed hospitalization. Among these AKI events, 379 were identified using the computerized algorithm, which achieved a sensitivity of 85.6%. In addition, of the 1108 patients who did not have AKI events during the indexed hospitalization, 13 were falsely defined as having AKI by the computerized algorithm, which achieved a specificity of 98.8%. Of the 64 false-negative (FN) AKI events missed by the computerized algorithm, 45 were not identified because of prolonged AKI or progression to CKD that began before the indexed hospitalization. The other 19 AKI events were missed by the computerized algorithm because SCr tests were performed too frequently within the 7 days before the highest SCr test during hospitalization, obscuring the real baseline SCr level and increasing the SCr level by less than 1.5 times. In contrast, the 13 false-positive (FP) AKI events mistaken by the computerized algorithm were exclusively due to fluctuating SCr levels caused by hemodialysis sessions (Table 3).

**Table 3:** The diagnostic table of the AKI events defined by the computerized algorithm.

Characteristics	AKI present	AKI absent	Total
Test positive	379 (TP)	13 (FP)	392
Test negative	64 (FN)	1095 (TN)	1159
Total	443	1108	1551
	Sensitivity: 85.6%	Specificity: 98.8%	

Cause analysis for FN AKI diagnosis			
Cause		Number of events/total events	Percentage of events
AKI events occurred in OPD		45/64	70.3%
Increases in creatinine was < 1.5 times compared to prior test		19/64	29.6%

Cause analysis for FP AKI diagnosis			
Cause		Number of events/total events	Percentage of events
Fluctuated serum creatinine levels caused by hemodialysis sessions		13/13	100%

AKI, acute kidney injury; TP, true positive; FP, false positive; FN, false negative; TN, true negative, OPD, outpatient department.

Statistical analysis was performed using chi square test.

### Diagnostic accuracy of computerized algorithm for CKD

To define the actual CKD cases in the present study, a complete series of all available previous eGFR records were reviewed by the researchers. Patients with eGFRs < 60 mL/min/1.73 m^2^ for > 90 days were defined as having CKD, criteria adapted from the KDIGO Clinical Practice Guideline for CKD. For those without baseline eGFR records within the previous 90 days, the latest eGFR was assumed to be the baseline. For patients with no prior eGFR records, the baseline eGFR was assumed to be normal.

Of the included 1551 patients, 1031 were defined as having CKD before the index hospitalization. Among these CKD cases, 976 were identified by the computerized algorithm, which achieved a sensitivity of 94.7%. The 55 FN CKD cases missed by the computerized algorithm due to the lack of previous eGFR records. In contrast, all 520 patients without CKD were correctly recognized using the computerized algorithm, which achieved a specificity of 100.0% (Table 4).

**Table 4:** The diagnostic table of the CKD events defined by the computerized algorithm.

Characteristics	CKD present	CKD absent	Total
Test positive	976 (TP)	0 (FP)	976
Test negative	55 (FN)	520 (TN)	575
Total	1031	520	1551
	Sensitivity: 94.7%	Specificity: 100.0%	

Cause analysis for FN CKD diagnosis			
Cause		Number of events/total events	Percentage of events
Existent CKD or ESRD without available medical records in the data base		55/55	100.0%

CKD, chronic kidney disease; TP, true positive; FP, false positive; FN, false negative; TN, true negative, ESRD, end-stage renal disease.

Statistical analysis was performed using chi square test.

### Comparison of accuracy between computerized algorithm and clinician’s diagnosis

**Fig. 2:**
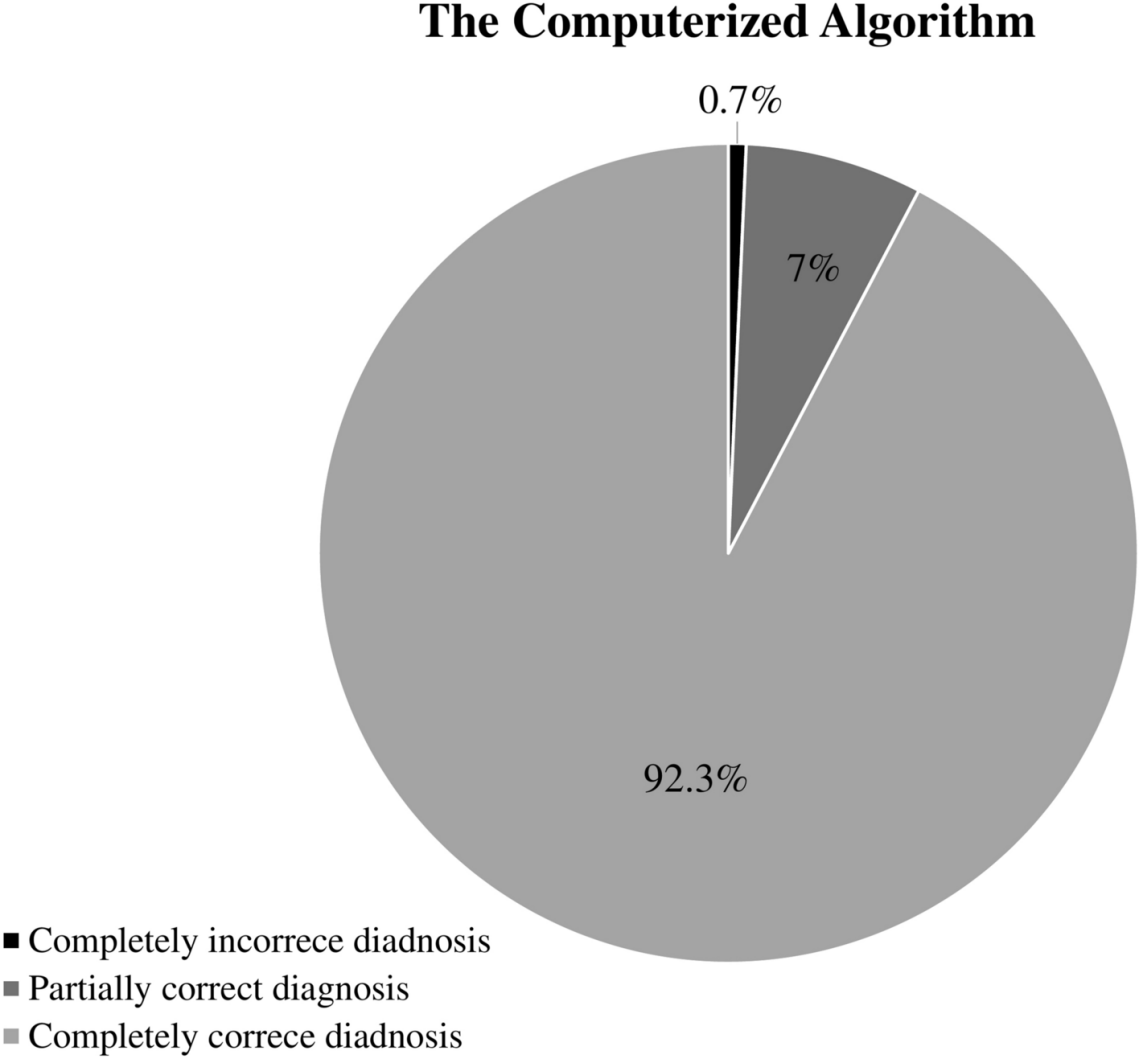
The accuracy of the Computerized Algorithm for renal impairment. Completely correct diagnosis was defined by correct diagnosis of both AKI and CKD; Partially correct diagnosis was defined by a single correct diagnosis of either AKI or CKD; Complete incorrect diagnosis was defined by incorrect diagnosis for both AKI and CKD. AKI, acute kidney injury; CKD, chronic kidney disease.

**Fig. 3:**
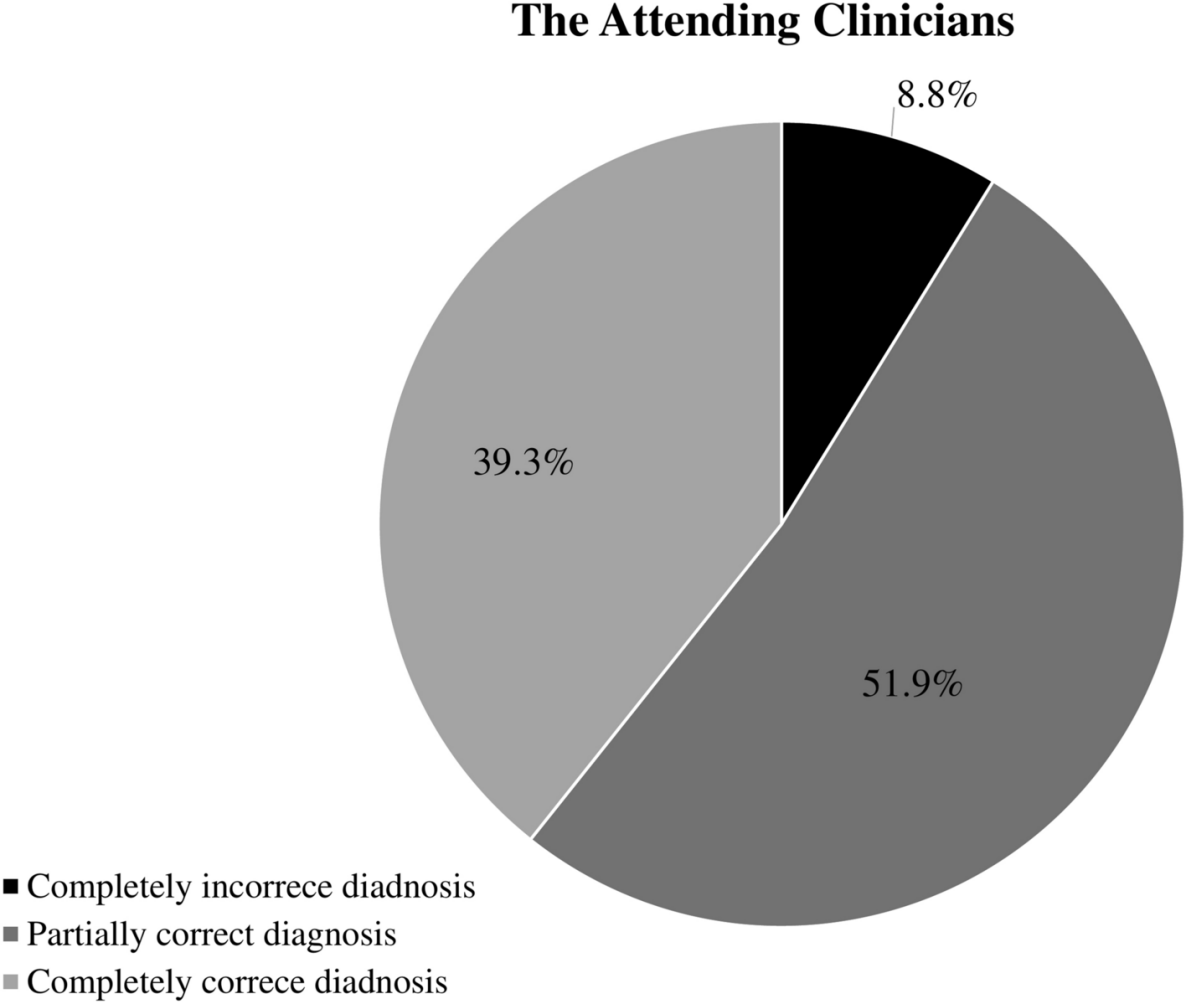
The accuracy of the attending clinicians for renal impairment. Completely correct diagnosis was defined by correct diagnosis of both AKI and CKD; Partially correct diagnosis was defined by a single correct diagnosis of either AKI or CKD; Complete incorrect diagnosis was defined by incorrect diagnosis for both AKI and CKD. AKI, acute kidney injury; CKD, chronic kidney disease.

To understand how the computerized algorithm improved the diagnostic accuracy of renal impairment, we compared its accuracy to that of the diagnoses made by the attending clinicians. In this analysis, correct diagnoses for both AKI and CKD in a single patient were regarded as “completely correct diagnoses”; correct diagnosis for either AKI or CKD with the other incorrectly diagnosed in a single patient was regarded as “partially correct diagnoses”; incorrect diagnoses for both AKI and CKD in a single patient were regarded as “completely incorrect diagnoses.” The computerized algorithm made a completely correct diagnosis in 92.3% (1431/1551) of the patients, a partially correct diagnosis in 7.0%, and a completely incorrect diagnosis in only 0.7% (12/1551) (Fig. 2). On the other hand, the attending clinicians made a completely correct diagnosis in only 39.3% (610/1551) of the patients and a partially correct diagnosis in 51.9% (805/1551). Surprisingly, in as many as 8.8% (136/1551) of the patients, the attending clinicians made a completely incorrect diagnosis (Fig. 3).

Furthermore, the diagnostic accuracies for AKI and CKD were compared separately to show the difference between the computerized algorithm’s accuracy for each and that of the attending clinicians. Regarding the accuracy of AKI, the computerized algorithm made an accurate diagnosis in 95.0% of the patients, which was significantly higher than the accuracy of the attending clinicians (95.0% vs. 57.0%, *P* < 0.001). Among medical patients (defined as patients admitted to service of physicians, n = 1100), the computerized algorithm made an accurate AKI diagnosis in 94.6% of patients, which was significantly higher than the accuracy of the attending clinicians (94.6% vs. 53.6%, *P* < 0.001). Among non-medical patients (defined as patients admitted to service of surgeons, n = 451), the computerized algorithm made an accurate AKI diagnosis in 96.2%, which was significantly higher than the accuracy of the attending clinicians (96.2% vs. 65.4%, *P* < 0.001). Regarding the diagnostic accuracy for CKD, the computerized algorithm achieved significantly higher accuracy than the attending clinicians (96.5% vs. 73.6%, *P* < 0.001). Among medical patients, the computerized algorithm made an accurate CKD diagnosis in 97.3%, which was significantly higher than the accuracy of the attending clinicians (97.3% vs. 76.0%, *P* < 0.001). Among non-medical patients, the computerized algorithm made an accurate CKD diagnosis in 94.5%, while the attending clinicians made an accurate diagnosis in 67.6%. Again, this difference was significant (*P* < 0.001) (Table 5). The performance of the computerized algorithm was assessed separately for patients from the ED and the OPD to determine the algorithm’s versatility. For the diagnosis of AKI, the accuracy of the computerized algorithm was significantly higher than that of the clinicians in both ED and OPD settings. Similarly, the algorithm demonstrated superior accuracy in diagnosing CKD compared with the clinicians. These results indicate that the computerized algorithm outperformed clinicians in various hospital settings (Table 6).

**Table 5:** The comparison of diagnostic accuracy between the Computerized Algorithm and the clinicians for renal impairment.

Characteristics	Computerized algorithm accuracy	Clinician’s accuracy	*P* value
AKI			
Total patients (n = 1551)	1474 (95.0%)	884(57.0%)	< .001
Medical patients (n = 1100)	1040 (94.6%)	589 (53.6%)	< .001
Non-medical patients (n = 451)	434 (96.2%)	295 (65.4%)	< .001
CKD			
Total patients (n = 1551)	1496 (96.5%)	1141 (73.6%)	< .001
Medical patients (n = 1100)	1070 (97.3%)	836 (76.0%)	< .001
Non-medical patients (n = 451)	426 (94.5%)	305 (67.6%)	< .001

AKI, acute kidney injury; CKD, chronic kidney disease.

Statistical analysis was performed using chi square method.

**Table 6:** The diagnostic accuracy of the Computerized Algorithm and the clinicians for renal impairment in patients from ED and OPD.

Characteristics	Computerized Algorithm accuracy	Clinician’s accuracy	*P* value
AKI			
Total patients (n = 1551)	1474 (95.0%)	884(57.0%)	< .001
ED (n = 878)	821(93.5%)	434(49.4%)	< .001
OPD (n = 673)	653(97.0%)	450(66.9%)	< .001
CKD			
Total patients (n = 1551)	1496 (96.5%)	1141 (73.6%)	< .001
ED (n = 878)	848(96.7%)	673(76.7%)	< .001
OPD (n = 673)	647(96.1%)	468(69.5%)	< .001

ED, emergency department; OPD, outpatient department; AKI, acute kidney injury; CKD, chronic kidney disease.

Statistical analysis was performed using chi square method.

## Discussion

The computerized algorithm used in the present study identified AKI events in hospitalized patients with a sensitivity of 85.6% and specificity of 98.8% and identified CKD in hospitalized patients with a sensitivity of 94.7% and specificity of 100.0%. The main causes of FN AKI cases were AKI occurring before the time of the indexed hospitalization and frequent SCr tests obscuring the increase in the highest SCr from its baseline. FN CKD was exclusively caused by the absence of past medical records. Despite these weaknesses, the computerized algorithm showed significant superiority over attending clinicians in terms of diagnostic accuracy for both AKI and CKD.

A major finding of the present study was that that both AKI events and CKD were underdiagnosed by the attending physicians and surgeons, which may have postponed appropriate management and referrals. Because we did not interview the clinicians individually, the reasons for this could not be explicitly explained. A possible explanation may be that, in cases in which a patient was not admitted because of AKI or was not managed by a nephrologist, minor or moderate increases in SCr that just fulfilled the AKI criteria may have been overlooked by the attending clinicians. Although an electronic AKI alert system is expected to improve the outcomes of patients with AKI, previous studies have reported inconsistent results. In the author’s opinion, two important tasks must be performed before an electronic AKI alert system can improve AKI outcomes. The first is the standardization of the methodology of electronic AKI alert systems to ensure that the alert is accurate and appropriate. The second is an algorithmic approach for the differential diagnosis of each specific case of AKI, which may provide personalized treatment instead of a unified AKI care bundle.

At a United Kingdom AKI consensus conference, experts recommended that, for the development of electronic AKI alert systems, a guide should be provided on how current diagnostic criteria should be applied and how baseline SCr levels should be selected^19^. Although many studies have investigated how electronic AKI alert systems change AKI outcomes, their diagnostic accuracy has seldom been reported. In a large-scale study involving 53,816 ED visits, Jonsson et al. tested the diagnostic accuracy of computerized algorithms applied to the original KDIGO AKI criteria and several modified criteria. The original KDIGO AKI criteria exhibited a sensitivity and specificity of 79% and 94%, respectively. However, the modified criteria that use the mean SCr of the last 30 days or 3–12 months may increase the sensitivity and specificity to 92–93% and 95–96%, respectively, which shows that the selection of baseline SCr levels is crucial for the diagnostic accuracy of electronic AKI alert systems^20^. In the present study, we aimed to standardize the methodology of electronic AKI alert systems so that they are applicable to smaller hospitals, which may encounter more cases without past SCr records. Regarding the diagnostic accuracy for AKI, the computerized algorithm used in the present study achieved a sensitivity of 85.6% and specificity of 98.0%. In contrast to the study by Jonsson et al. referred to above, we did not use the mean SCr of the past period, but the last SCr value as the baseline SCr level. As the mean SCr may be confounded by episodes of fully recovered AKI, the last SCr record is a more reasonable baseline. Notably, we also attempted to use the first abnormal SCr level for the computerized algorithm to define the occurrence of AKI in this study, but it resulted in lower sensitivity. This was because the first abnormal SCr level may not have been as high as 1.5 times of its prior reading, and the AKI event of the higher SCr test that occurred later was neglected. Thus, in the present study, the highest SCr level during hospitalization was used by the computerized algorithm to define AKI. Nonetheless, in the context of real-time renal monitoring, AKI events are eventually detected as SCr increases to > 1.5 times its previous value.

In addition to diagnosing AKI events, the computerized algorithm was used to identify CKD cases. Unlike the management strategies for AKI, CKD requires early diagnosis and appropriate referral to specialists^21^. Several electronic tools with various diagnostic algorithms have been developed for the early diagnosis of CKD. Approaches to diagnostic algorithms include identifying diagnostic codes^22–25^ and checking eGFR laboratory criteria^26^ with or without consideration of proteinuria^24,25^. The sensitivities and specificities of these algorithms range from 87 to 100% and 96–99%, respectively, demonstrating the high accuracy of CKD diagnostic algorithms on average^27^. In the present study, although the computerized algorithm used only eGFR laboratory criteria, it achieved a sensitivity and specificity of 94.7% and 100.0%, respectively. Notably, all FN CKD cases were missed due to the absence of past laboratory data. The results show that the accuracy of the computerized algorithm for CKD in the present study is acceptable.

While the underdiagnosis or misdiagnosis of AKI by clinicians is an issue that needs to be emphasized, the present study objectively shows its significance. A major cause of this situation may be that minor fluctuations in SCr level are considered prerenal AKI, which is a sign of a changed volume status and is less likely to adversely affect outcomes^28^. Therefore, the diagnosis of AKI may be neglected. Nonetheless, such minor and evanescent increases in SCr may progress to intrinsic structural AKI and cause adverse outcomes^29–31^. To address this, computerized algorithms may alert clinicians to pay attention to minor but important changes in SCr levels and initiate appropriate management. A major cause of the underdiagnosis of CKD may be that the current definition of CKD is controversially considered to cause overdiagnosis in some cases^32^. For example, elderly female patients with an eGFR of 50–59 mL/min/1.73 m^2^ without proteinuria are considered to have CKD stage 3a according to the current definition. For many nephrologists, such low eGFR values are considered a normal age-related decline rather than true CKD^33^. This may contribute to the reason patients with CKD are underdiagnosed by clinicians.

Different applications of the KDIGO criteria can yield varying AKI incidences. A 2020 post hoc analysis tested 30 methods using SCr, urine output, and their combination and found an AKI incidence ranging from 28 to 75%^34^. In this study, the lowest incidence occurred when urine output was not considered, suggesting that the present study, which was based only on SCr, may underestimate the incidence of AKI. However, measuring urine output requires at least 6 h, which limits the point-of-care decisions required in the present study.

Although AKI alert systems have been tested in many studies, most have reported higher AKI documentation rates, while their accuracy has rarely been mentioned^35–37^. In a randomized controlled trial testing the value of an electronic AKI alert system, the sensitivity and specificity were reported to be as high as 99.8% and 97.7%, respectively, which are higher than those in the present study.^38^ This supports the finding that our computerized algorithm has better diagnostic accuracy than clinicians.

In the near future, we intend to implement the program as a Clinical Decision Support System (CDSS) for our existing health record viewers. This will enable clinicians to receive real-time alerts for potential AKI and CKD and may improve the early diagnosis of renal impairment. The goal is to provide a tool for more accurate and timely identification of renal impairment, which can lead to improved patient outcomes.

The limitations of the present study include its retrospective design; its accuracy remains to be validated in another prospective study; a relatively small number of cases in which its application needs to be further tested; and a lack of interventions that may truly improve patient outcomes. To compensate for these limitations, we will soon incorporate an algorithmic approach for differential diagnosis and a decision guide into the computerized algorithm and investigate how this changes the outcomes of AKI in a trial. Additionally, the present single-center approach limits the extension of our findings to other centers. Another limitation is that patients with ESRD could not be automatically excluded using the computerized algorithm. Therefore, fluctuating SCr levels due to hemodialysis treatment may be falsely interpreted as AKI events, and higher SCr levels may be observed in the non-AKI group. This limitation may be compensated by avoid SCr test on after dialysis sessions. For patients without previous SCr test results, the baseline SCr level was assumed to be normal to avoid missing treatable AKI events. Although stationary renal function in some patients with CKD may be falsely classified as an AKI event, this approach is relatively conservative.

In conclusion, we developed a simple and easy-to-construct computerized algorithm for diagnosis of renal impairment. The computerized algorithm improved the diagnostic accuracy for AKI and CKD, which may lead to the earlier diagnosis and appropriate management of renal impairment. In the future, an algorithmic approach for the differential diagnosis of AKI and a decision guide may be incorporated into this system to achieve higher decision aid capability.

## Data Availability

The datasets used and/or analyzed during the current study available from the corresponding author on reasonable request.

## Abbreviations

AKI: Acute kidney injury
CKD: Chronic kidney disease
ED: Emergency department
eGFR: Estimated glomerular filtration rate
ESRD: End-stage renal disease
FN: False negative
FP: False positive
ICU: Intensive care unit
SCr: Serum creatinine
TN: True negative
TP: True positive
